# Statistical Parametric Mapping to Identify Differences between Consensus-Based Joint Patterns during Gait in Children with Cerebral Palsy

**DOI:** 10.1371/journal.pone.0169834

**Published:** 2017-01-12

**Authors:** Angela Nieuwenhuys, Eirini Papageorgiou, Kaat Desloovere, Guy Molenaers, Tinne De Laet

**Affiliations:** 1 Department of Rehabilitation Sciences, KU Leuven, Leuven, Belgium; 2 Clinical Motion Analysis Laboratory, University Hospitals Leuven, Leuven, Belgium; 3 Department of Development and Regeneration, KU Leuven, Leuven, Belgium; 4 Department of Orthopedics, University Hospitals Leuven, Leuven, Belgium; 5 Faculty of Engineering Science, KU Leuven, Leuven, Belgium; Boston Children's Hospital / Harvard Medical School, UNITED STATES

## Abstract

Experts recently identified 49 joint motion patterns in children with cerebral palsy during a Delphi consensus study. Pattern definitions were therefore the result of subjective expert opinion. The present study aims to provide objective, quantitative data supporting the identification of these consensus-based patterns. To do so, statistical parametric mapping was used to compare the mean kinematic waveforms of 154 trials of typically developing children (n = 56) to the mean kinematic waveforms of 1719 trials of children with cerebral palsy (n = 356), which were classified following the classification rules of the Delphi study. Three hypotheses stated that: (a) joint motion patterns with ‘no or minor gait deviations’ (n = 11 patterns) do not differ significantly from the gait pattern of typically developing children; (b) all other pathological joint motion patterns (n = 38 patterns) differ from typically developing gait and the locations of difference within the gait cycle, highlighted by statistical parametric mapping, concur with the consensus-based classification rules. (c) all joint motion patterns at the level of each joint (n = 49 patterns) differ from each other during at least one phase of the gait cycle. Results showed that: (a) ten patterns with ‘no or minor gait deviations’ differed somewhat unexpectedly from typically developing gait, but these differences were generally small (≤3°); (b) all other joint motion patterns (n = 38) differed from typically developing gait and the significant locations within the gait cycle that were indicated by the statistical analyses, coincided well with the classification rules; (c) joint motion patterns at the level of each joint significantly differed from each other, apart from two sagittal plane pelvic patterns. In addition to these results, for several joints, statistical analyses indicated other significant areas during the gait cycle that were not included in the pattern definitions of the consensus study. Based on these findings, suggestions to improve pattern definitions were made.

## Introduction

Three-dimensional gait analysis (3DGA) serves as a golden standard to objectively evaluate pathological gait in children with cerebral palsy (CP) and it has been shown to alter clinical decision making and improve treatment outcome [1–4]. However, the clinical interpretation of kinematic and kinetic gait data is subjective and therefore less reliable [5]. To support the clinical understanding of gait data, many attempts have been made to recognize different gait patterns from kinematic and kinetic reports, using either qualitative or quantitative approaches [6–13]. Regarding quantitative approaches, complex clinical interpretation of the patterns hinders the applicability of the classifications in medical practice. Qualitative approaches have also been criticized for unclear pattern definitions and lack of transparency in the construction of the classification [6]. In an attempt to overcome some of these methodological challenges, gait patterns for the different lower limb joints have recently been proposed for children with CP following an international consensus study [11]. Based on the judgment of an expert panel and supported by literature, 49 gait patterns were defined for the following joints: pelvis in the sagittal (n = 6), coronal (n = 4), and transverse (n = 4) plane; hip in the sagittal (n = 3), coronal (n = 4), and transverse (n = 3) plane; knee in the sagittal plane during stance (n = 7) and swing (n = 6) phase; ankle in the sagittal plane during stance (n = 5) and swing (n = 4) phase; foot progression angle (n = 3). The pattern definitions or classification rules on which consensus was achieved, were based on kinematic descriptions of locations within the gait cycle that deviate from the gait pattern of typically developing (TD) children. To a lesser extent, pattern definitions also included kinetic abnormalities for the hip patterns and for the knee patterns during stance in the sagittal plane. Precise pattern definitions are available in the supplementary material (S1 Table).

The pattern definitions were the result of an informed, yet subjective opinion of an expert panel and therefore may provide an incomplete picture on joint patterns during gait in CP. Hence, the content validity of the classification could be threatened and objective, quantitative data should be provided to support the identification of these consensus-based patterns. To this end, the present study uses statistical parametric mapping (SPM), a statistical approach which allows hypothesis testing on kinematic and kinetic waveforms without the need of a priori data reduction [14]. SPM is used to analyze kinematic and kinetic gait trials that were classified according to the definitions of the consensus study, in a large cohort of children with CP. If the classification has good content validity, all clinically relevant gait deviations should be included in the pattern definitions and all ambulatory children with spastic CP should be classifiable by a clinician, fitting the classification rules. Joint kinematics that do not fit any pathological pattern, should therefore be classified as having ‘no or minor gait deviations’, which is a pattern that was defined at the level of each joint [11]. As a result, **the first hypothesis** stated that all kinematic trials classified as ‘no or minor gait deviations’ do not differ significantly from the gait pattern of TD children. **The second hypothesis** stated that kinematic and kinetic trials classified as pathological joint motion patterns, are significantly different from the gait of TD children and that the locations of difference within the gait cycle, which are highlighted by SPM, concur with the locations described in the classification rules of the consensus study [11]. A confirmation of this second hypothesis provides evidence for the feasibility of developing algorithms for automatic classification (e.g. Bayesian networks [13], [15]) and for the classes and classification rules of the consensus study [11]. In light of this, **the third hypothesis** stated that the pathological patterns at the level of each joint differ from each other during at least one phase of the gait cycle.

## Materials and Methods

### Patient group

A sample of convenience was selected from the database of the clinical motion analysis laboratory of University Hospital Pellenberg. The database was searched for gait analysis sessions of children with unilateral or bilateral spastic CP, aged between 3 to 18 years and GMFCS level I, II, or III. All gait analysis sessions which were obtained for research or clinical purposes between November 2001 and August 2015, were eligible to be included. Children with marked signs of dystonia or ataxia were excluded. Children who had undergone previous treatment were eligible to be included in the study, as one of the goals for the joint motion patterns was that they could be used, among others, to evaluate changes in the gait pattern of patients over time. In total, 459 gait analysis sessions corresponding to 356 CP patients were included of which 154 sessions were post-treatment. On average, patients were evaluated 60 days post-Botulinum toxin type A injections, 392 days after single event multilevel surgery, and 375 days after selective dorsal rhizotomy. One gait analysis session was available for 275 patients; two sessions were available for 67 patients, and three to six sessions for 14 patients. To compare pathological gait to the normal gait pattern, the reference database of the hospital was used, which consisted of 56 TD children between 5 to 18 years old, with no history of musculoskeletal or neuromotor disorders.

### Data collection

Standardized 3DGA measurements were performed using ten to fifteen optoelectronic cameras (Vicon Motion Systems, Oxford, UK) and two force platforms (Advanced Mechanical Technology Inc., USA), which were embedded in a 10m walkway. Reflective markers were fixed on anatomical landmarks according to the Plug-In-Gait model and all children were asked to walk barefoot and at a self-selected speed. Nexus software was used to estimate gait cycles, joint angles, and joint moments, which were normalized to body mass. Kinematic and kinetic waveforms were also time-normalized to the gait cycle, or to stance and swing phase when appropriate. Each waveform was interpolated to intervals of 2%, yielding a total of 51 data points per curve. Subsequently, these kinematic and kinetic trials were imported into a custom-made Matlab^®^ software tool. Trials with artifacts or with signs of inaccurate marker placement were excluded. To this end, the range of motion and position of the knee varus-valgus angle was evaluated[16]. Outliers or trials that were not representing a child’s gait pattern were also excluded. Outliers were determined based on video observation in combination with visual inspection of the waveform (i.e. when the distance from the trial of a patient to the average of all trials of that patient was larger than two standard deviations of the TD database). For each TD child, two to four good quality trials of the left or right side were included for SPM analysis. In total, 154 good quality kinematic trials were included, of which 148 trials also included kinetic data. For patients with CP, a total of 1719 good quality kinematic trials were available and were included in the study. Of these trials, 985 also had kinetic data available. A median of 3 trials (interquartile range 2 tot 7) were available for classification per patient per side. The maximum number of trials for each patient and TD child was included because the research question of this methodological study concerns the analysis of differences between kinematic and kinetic groups as they are defined subjectively by clinicians, irrespective of whether trials belong to the same patient or different patients, unlike for an analysis of the prevalence of the joint motion patterns within different patient groups, which would require a fixed number of trials (or an averaged trial) per patient. Following the definitions of the consensus study [11], each included trial was classified by a clinical expert rater (one of two raters) for the following joints: pelvis in the sagittal (PS), coronal (PC), and transverse (PT) plane; hip in the sagittal (HS), coronal (HC), and transverse (HT) plane; knee during stance (KSTS) and during swing (KSWS) in the sagittal plane; ankle during stance (ASTS) and during swing (ASWS) in the sagittal plane, and foot progression angle (FPA). A brief description of each pattern (n = 49) is presented in Table 1; full descriptions are available in the supplementary material (S1 Table).

**Table 1:** Observed frequency (in %, N = 1719 trials) and brief description of all sagittal, coronal, and transverse plane joint motion patterns defined during the consensus study.

	(%)
**SAGITTAL PLANE**	
**Pelvis**	
PS0—Normal pelvic motion/posture—no or minor gait deviations	16.0
PS1—Increased range of motion	29.6
PS2—Increased anterior tilt on average	16.1
PS3—Increased anterior tilt and increased range of motion	35.9
PS4—Decreased anterior tilt (posterior tilt)	1.3
PS5—Decreased anterior tilt (posterior tilt) and increased range of motion	1.1
**Hip**	
HS0—Normal hip motion—no or minor gait deviations	55.3
HS1—Hip extension deficit	27.6
HS2—Continuous excessive hip flexion	17.1
**Knee during stance**	
KSTS0—Normal knee motion during stance—no or minor gait deviations	14.8
KSTS1—Increased knee flexion at initial contact	7.3
KSTS2—Increased knee flexion at initial contact and earlier knee extension movement	20.7
KSTS3—Knee hyperextension	8.1
KSTS4—Knee hyperextension and increased knee flexion at initial contact	10.9
KSTS5—Increased flexion in midstance and internal flexion moment present	23.0
KSTS6—Increased flexion in midstance and internal extension moment present	15.1
**Knee during swing**	
KSWS0—Normal knee motion during swing—no or minor gait deviations	35.4
KSWS1—Delayed peak knee flexion	21.5
KSWS2—Increased peak knee flexion	12.6
KSWS3—Increased and delayed peak knee flexion	9.4
KSWS4—Decreased peak knee flexion	10.8
KSWS5—Decreased and delayed peak knee flexion	10.3
**Ankle during stance**	
ASTS0—Normal ankle motion during stance—no or minor gait deviations	38.6
ASTS1—Horizontal second ankle rocker	28.0
ASTS2—Reversed second ankle rocker	9.4
ASTS3—Equinus gait	4.2
ASTS4—Calcaneus gait	19.7
**Ankle during swing**	
ASWS0—Normal ankle motion during swing—no or minor gait deviations	40.0
ASWS1—Insufficient prepositioning in terminal swing	6.5
ASWS2—Continuous plantarflexion during swing (drop foot)	18.7
ASWS3—Excessive dorsiflexion during swing	34.8
**CORONAL PLANE**	
**Pelvis**	
PC0—Normal pelvic motion/posture—no or minor gait deviations	48.6
PC1—Increased pelvic range of motion	29.1
PC2—Continuous pelvic elevation	11.8
PC3—Continuous pelvic depression	10.6
**Hip**	
HC0—Normal hip motion—no or minor gait deviations	62.9
HC1—Excessive hip abduction in swing	21.6
HC2—Continuous excessive hip abduction	9.2
HC3—Continuous excessive hip adduction	6.3
**TRANSVERSE PLANE**	
**Pelvis**	
PT0—Normal pelvic motion/posture—no or minor gait deviations	44.4
PT1—Increased pelvic range of motion	30.4
PT2—Excessive pelvic external rotation during the gait cycle	13.0
PT3—Excessive pelvic internal rotation during the gait cycle	12.2
**Hip**	
HT0—Normal hip motion—no or minor gait deviations	75.4
HT1—Excessive hip external rotation during the gait cycle	8.9
HT2—Excessive hip internal rotation during the gait cycle	15.7
**Foot**	
FPA0—Normal foot progression angle—no or minor gait deviations	66.6
FPA1—Outtoeing	15.7
FPA2—Intoeing	17.7

Described deviations such as increased or excessive joint angles refer to deviations which are more than one standard deviation away from the TD reference database. A more detailed description of the patterns is available in the supplementary material (S1 Table).

### Statistical analysis

To test the first and second hypothesis, SPM unpaired t-tests were performed, comparing the mean kinematic (or kinetic) angle of each pattern to the respective mean kinematic (or kinetic) angle of the TD group (α = 0.01). For the third hypothesis, an SPM one-way-ANOVA was performed to examine whether the mean joint angles of the patterns per joint differed significantly from each other (α = 0.01).

**Fig 1:**
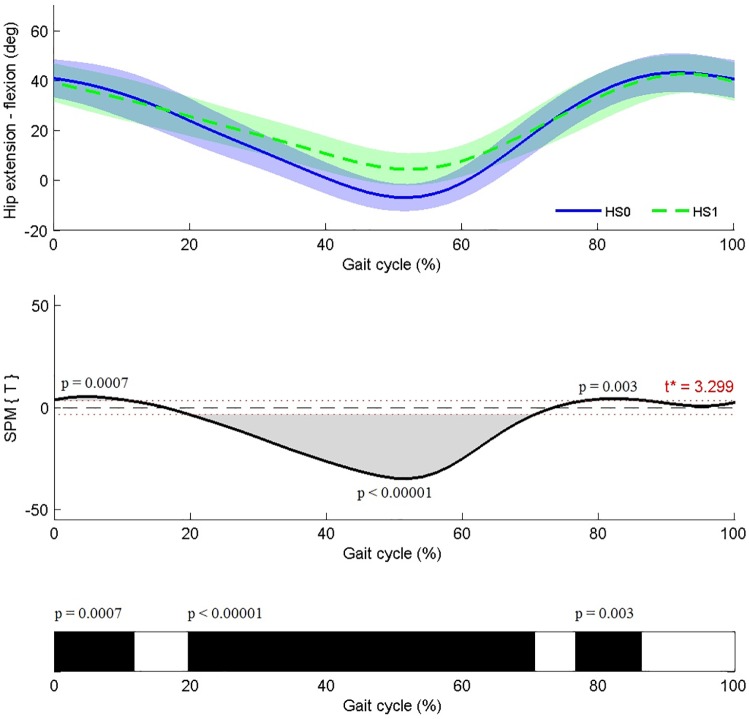
Example of summarized presentation of SPM results. Upper graph shows the mean kinematic hip angle in the sagittal plane of trials classified as ‘no or minor gait deviations’ (HS0) or ‘hip extension deficit’ (HS1). Middle graph shows SPM {t} statistic as a function of the gait cycle. The critical threshold (t*) was exceeded between 0–12%, 20–71%, and 76–86% of the gait cycle. Lower black bars represent a simplified visualization of the significant areas indicated by the SPM{t} statistic.

For each SPM ANOVA or t-test, a statistical parametric map (SPM{F} or SPM{t} respectively) was created by calculating the conventional univariate t- or F-statistic at each point of the gait curve [14]. Afterwards, Random Field Theory allowed an estimation of the critical threshold above which only 1% (α = 0.01) of equally smooth random data was expected to cross [17]. If the SPM{F} crossed the critical threshold, post-hoc SPM{t} maps were calculated for between-group comparisons. If at any time, an SPM{t} crossed the critical threshold, a supra-threshold cluster was created, indicating a significant difference between two joint motion patterns in a specific location of the gait cycle. A Bonferroni correction was applied for each joint to adjust α for multiple post-hoc comparisons. For each supra-threshold cluster, the probability (p-value) of discovering a cluster with similar proportions when testing equally smooth random data was calculated [17]. Because of the high number of statistical analyses, the SPM results are presented in a summarized manner. Instead of SPM{t} curves, black bars will be shown, indicating the locations within the gait cycle during which a supra-threshold cluster was identified (Fig 1). Taking into account previously reported measurement errors that are inherent to 3DGA, a significant difference was interpreted as relevant if the mean waveforms were at least 3° removed from each other within the areas of significance as indicated by the SPM output (i.e. black bars) [16], [18]. All analyses were performed on retrospectively collected and anonymized patient data, using open-source SPM1d code (vM.01.0003; www.spm1D.org) in Matlab^®^. The study was approved by the Medical Ethical Committee of University Hospitals Leuven (s56036).

## Results

Table 2 describes the characteristics of patients with CP and TD children.

**Table 2:** Demographic characteristics of CP (n = 356) and TD (n = 56) children.

	CP (n)	TD (n)
**Gender**		
Male	212	24
Female	144	32
**Weight (mean (SD), in kg)**	32.2 (14.0)	40.1 (17.7)
**Height (mean (SD), in m)**	1.34 (0.20)	1.48 (0.21)
**Diagnosis**		
Bilateral CP	219	
Unilateral CP	137	
**GMFCS**		
Level I	192	
Level II	117	
Level III	47	
**Number of 3DGA sessions**	459	56
**Age at time of 3DGA (mean (SD), in years)**	9 years, 10 months (3 years, 6 months)	11 years, 1 month (3 years, 10 months)

SD = standard deviation

### Hypothesis 1: Kinematic trials classified as ‘no or minor gait deviations’ at the level of each joint do not differ significantly from the gait pattern of TD children

**Fig 2:**
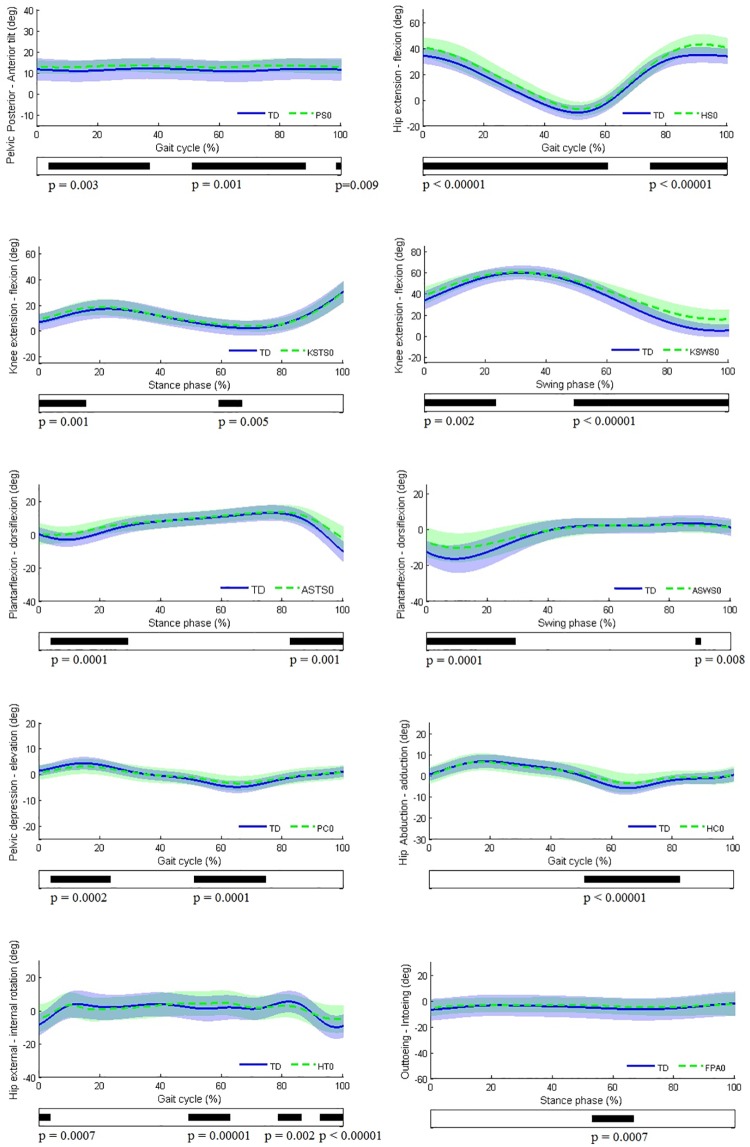
Each graph shows the mean kinematic angle of TD gait versus the pattern ‘no or minor gait deviations’ at the level of each joint, except for the pelvis in the transverse plane (no significant differences). Black bars indicate gait phases during which the SPM{t} statistic exceeded the critical threshold.

The pattern with ‘no or minor gait deviations’ differed significantly from TD gait during at least one phase of the gait cycle for the foot progression angle, the ankle and knee during stance and swing phase in the sagittal plane, the hip in the sagittal, coronal, and transverse plane, and the pelvis in the sagittal and coronal plane (all p<0.01; Fig 2). Only for the pattern of the pelvis in the transverse plane, no significant differences were identified (Fig 2).

In the locations of the gait cycle where significant deviations from TD gait were identified, the differences between the mean kinematic angles were generally small (≤3°) (Fig 2). Larger relevant deviations (≥3°) from TD gait were identified for the sagittal joint pattern ‘no or minor gait deviations’ of the **hip, knee during swing, and ankle during stance and swing phase**.

With respect to the **hip**, markedly increased flexion was noted between 0–61% and 74–100% of the gait cycle (both p<0.00001) compared to TD gait, while for the **knee during swing**, the flexion angle was increased between 0–24% and 49–100% of swing (p = 0.00227 and p<0.00001 respectively) At the level of the **ankle**, the patterns ‘no or minor gait deviations’ (ASTS0 and ASWS0) showed markedly increased dorsiflexion during push-off (82–100% of stance, p = 0.0013) and during the first 29% of swing (p<0.0001). A slight increase in dorsiflexion was also identified between 4–29% of stance phase (p<0.0001).

### Hypothesis 2: kinematic and kinetic trials classified as one of the pathological joint motion patterns are significantly different from TD gait; locations of difference within the gait cycle that are highlighted by SPM concur with the locations described in the classification rules of the consensus study

**Fig 3:**
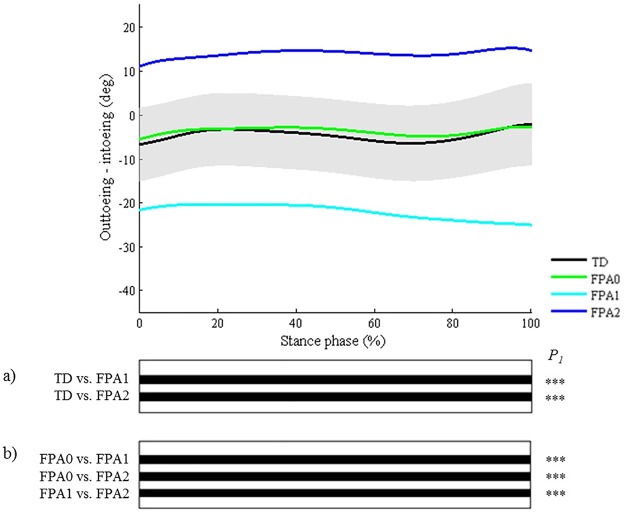
Foot progression angle (FPA). Top graph shows the mean kinematic angle of TD gait and of each consensus-based pattern of the foot progression angle. Black bars indicate significant gait phases during which the SPM{t} statistic exceeded the critical threshold. Panel (**a**) shows results of hypothesis 2 (i.e. unpaired t-tests, α = 0.01); panel (**b**) shows results of hypothesis 3 (i.e. post-hoc unpaired t-tests, α = 0.003). * p<0.01, ** p<0.001, *** p<0.00001. P_1_ indicates the p-value of the first cluster during the stance phase.

**Fig 4:**
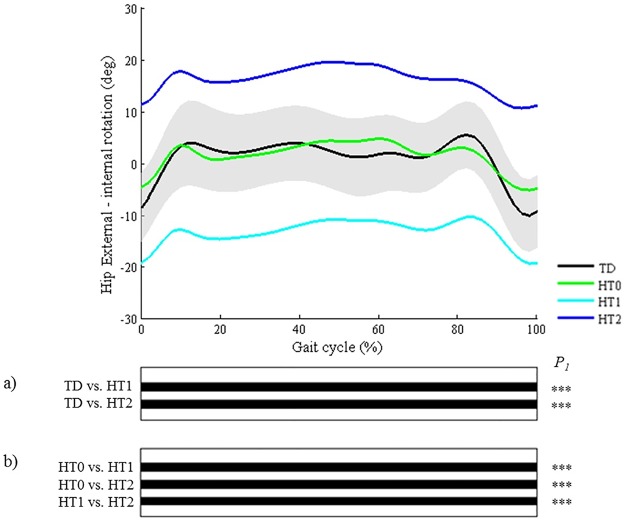
Hip in the transverse plane (HT). Top graph shows the mean kinematic angle of TD gait and of each consensus-based pattern of the hip in the transverse plane. Black bars indicate significant gait phases during which the SPM{t} statistic exceeded the critical threshold. Panel (**a**) shows results of hypothesis 2 (i.e. unpaired t-tests, α = 0.01); panel (**b**) shows results of hypothesis 3 (i.e. post-hoc unpaired t-tests, α = 0.003). * p<0.01, ** p<0.001, *** p<0.00001. P_1_ indicates the p-value of the first cluster during the gait cycle.

**Fig 5:**
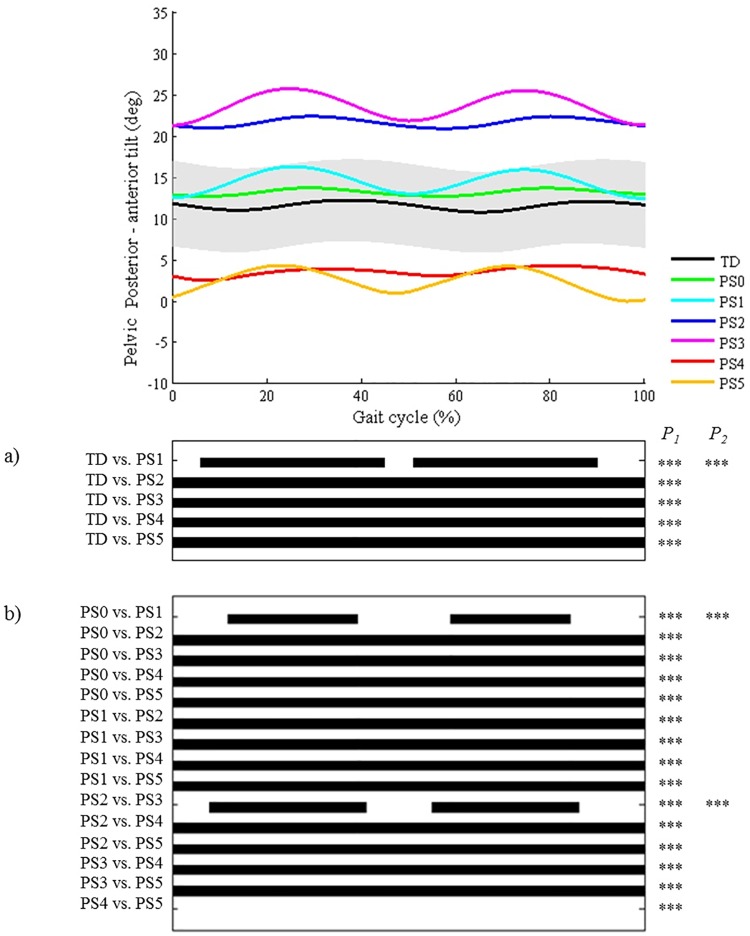
Pelvis in the sagittal plane (PS). Top graph shows the mean kinematic angle of TD gait and of each consensus-based pattern of the pelvis in the sagittal plane. Black bars indicate significant gait phases during which the SPM{t} statistic exceeded the critical threshold. Panel (**a**) shows results of hypothesis 2 (i.e. unpaired t-tests, α = 0.01); panel (**b**) shows results of hypothesis 3 (i.e. post-hoc unpaired t-tests, α = 0.0006). * p<0.01, ** p<0.001, *** p<0.00001. P_1_ indicates the p-value of the first cluster during the gait cycle, P_2_ the second cluster.

**Fig 6:**
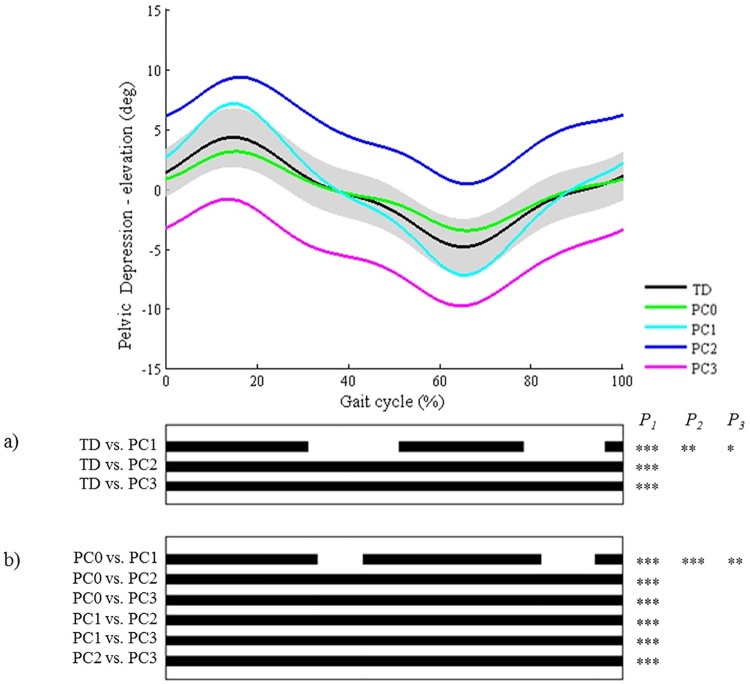
Pelvis in the coronal plane (PC). Top graph shows the mean kinematic angle of TD gait and of each consensus-based pattern of the pelvis in the coronal plane. Black bars indicate significant gait phases during which the SPM{t} statistic exceeded the critical threshold. Panel (**a**) shows results of hypothesis 2 (i.e. unpaired t-tests, α = 0.01); panel (**b**) shows results of hypothesis 3 (i.e. post-hoc unpaired t-tests, α = 0.002). * p<0.01, ** p<0.001, *** p<0.00001. P_1_ indicates the p-value of the first cluster during the gait cycle, P_2_ the second cluster, etc.

**Fig 7:**
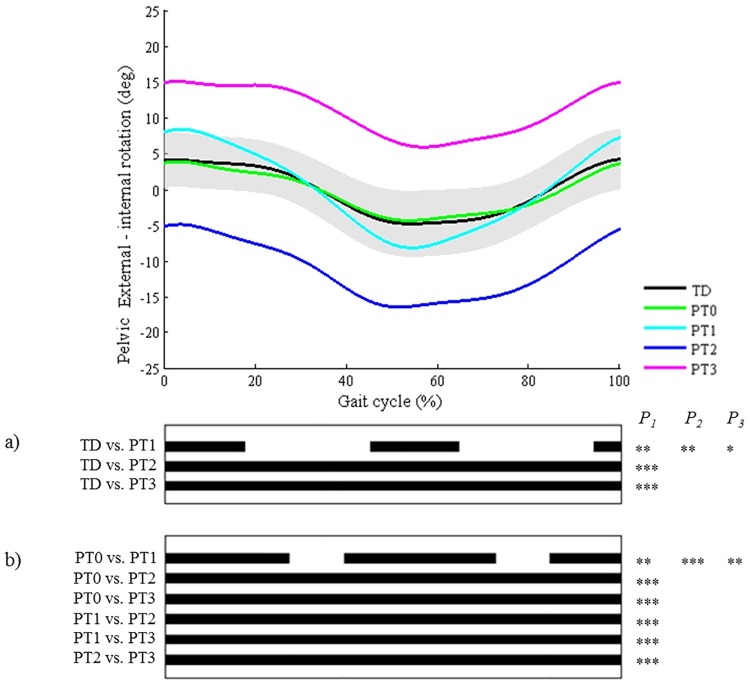
Pelvis in the transverse plane (PT). Top graph shows the mean kinematic angle of TD gait and of each consensus-based pattern of the pelvis in the transverse plane. Black bars indicate significant gait phases during which the SPM{t} statistic exceeded the critical threshold. Panel (**a**) shows results of hypothesis 2 (i.e. unpaired t-tests, α = 0.01); panel (**b**) shows results of hypothesis 3 (i.e. post-hoc unpaired t-tests, α = 0.002). * p<0.01, ** p<0.001, *** p<0.00001. P_1_ indicates the p-value of the first cluster during the gait cycle, P_2_ the second cluster, etc.

All pathological patterns differed significantly from TD gait, on average throughout 91% of the gait cycle (or of stance/swing phase regarding the patterns of the foot progression angle and the knee and ankle joint in the sagittal plane). Locations of difference that were highlighted by SPM concurred with the locations described in the classification rules for all joint motion patterns of the following joints: foot progression angle, hip in the transverse plane, and pelvis in the sagittal, coronal, and transverse plane (Figs 3–7). Out of all joint motion patterns of these five joints (n = 20), SPM analysis highlighted small (≤3°) differences from TD gait only for the patterns ‘increased pelvic range of motion’ in the coronal (PC1) and transverse (PT1) plane. For the other eighteen pathological joint motion patterns, large significant differences (>3°) with the mean TD pattern were identified.

**Fig 8:**
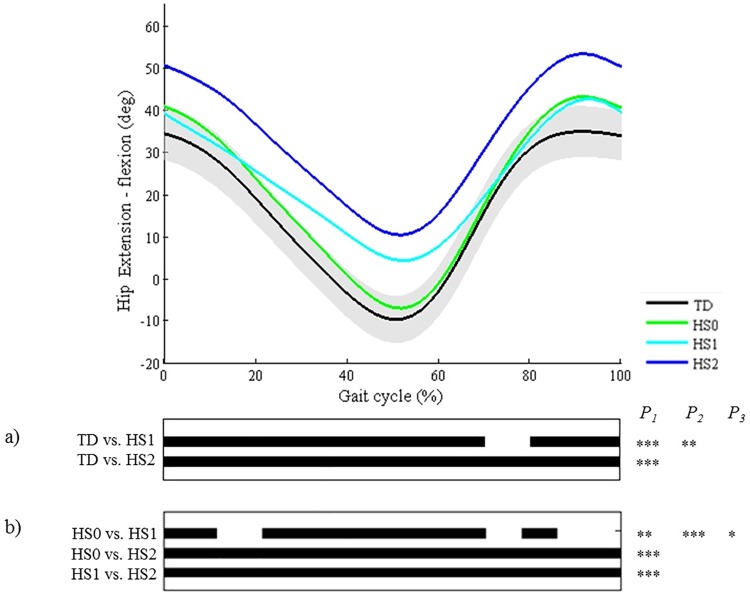
Hip in the sagittal plane (HS). Top graph shows the mean kinematic angle of TD gait and of each consensus-based pattern of the hip in the sagittal plane. Black bars indicate significant gait phases during which the SPM{t} statistic exceeded the critical threshold. Panel (**a**) shows results of hypothesis 2 (i.e. unpaired t-tests, α = 0.01); panel (**b**) shows results of hypothesis 3 (i.e. post-hoc unpaired t-tests, α = 0.003). * p<0.01, ** p<0.001, *** p<0.00001. P_1_ indicates the p-value of the first cluster during the gait cycle, P_2_ the second cluster, etc.

**Fig 9:**
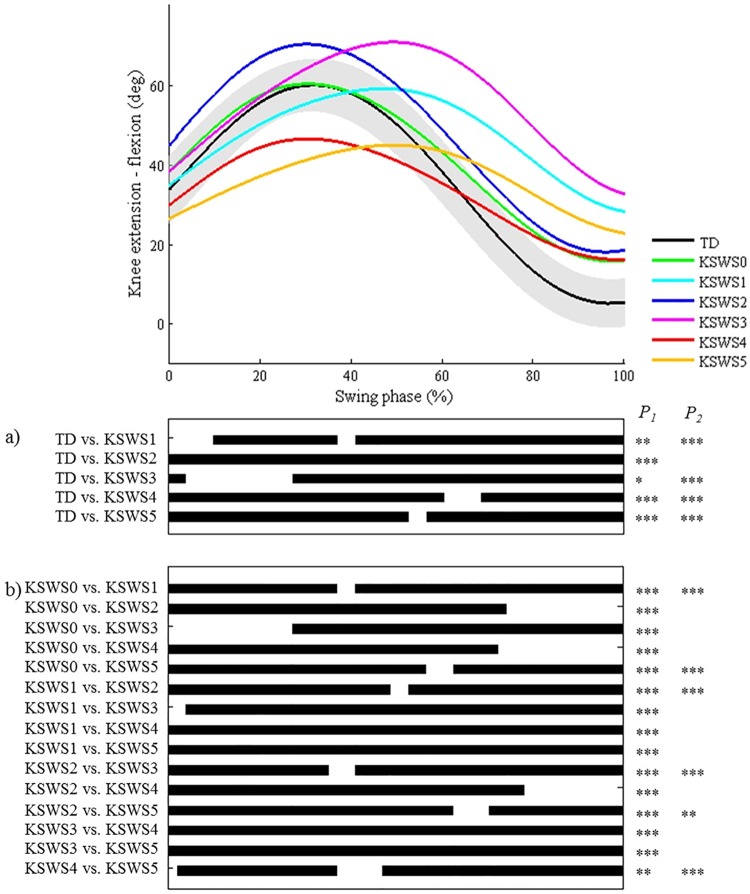
Knee during swing in the sagittal plane (KSWS). Top graph shows the mean kinematic angle of TD gait and of each consensus-based pattern of the knee during swing in the sagittal plane. Black bars indicate significant gait phases during which the SPM{t} statistic exceeded the critical threshold. Panel (**a**) shows results of hypothesis 2 (i.e. unpaired t-tests, α = 0.01); panel (**b**) shows results of hypothesis 3 (i.e. post-hoc unpaired t-tests, α = 0.0006). * p<0.01, ** p<0.001, *** p<0.00001. P_1_ indicates the p-value of the first cluster during the swing phase, P_2_ the second cluster.

**Fig 10:**
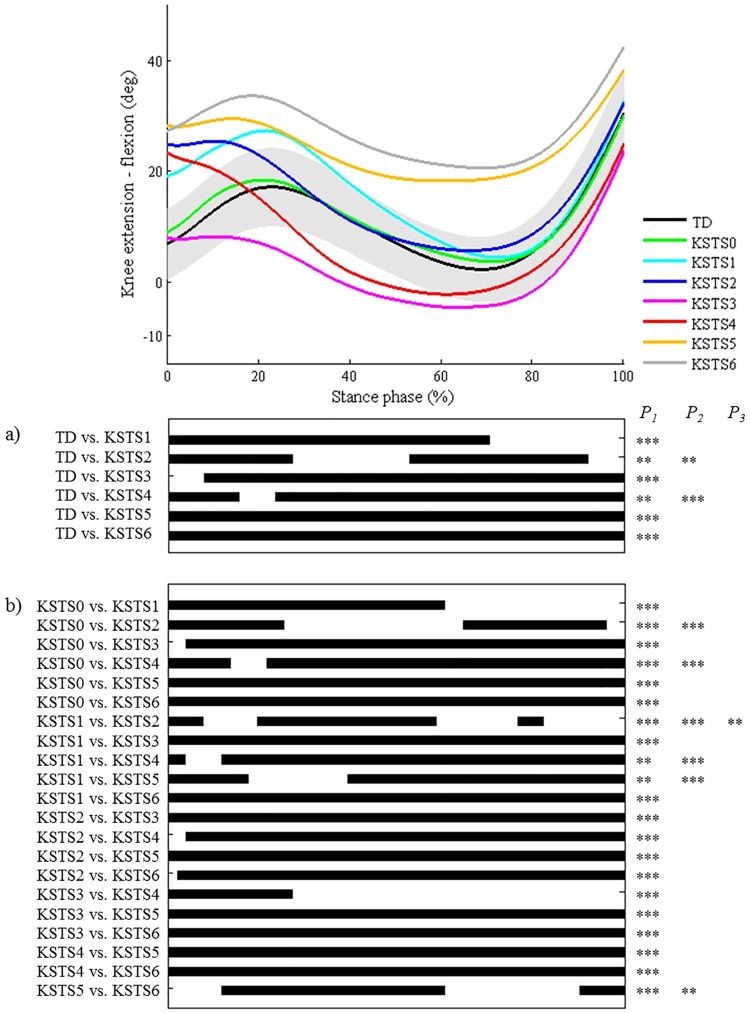
Knee during stance in the sagittal plane (KSTS). Top graph shows the mean kinematic angle of TD gait and of each consensus-based pattern of the knee during stance in the sagittal plane. Black bars indicate significant gait phases during which the SPM{t} statistic exceeded the critical threshold. Panel (**a**) shows results of hypothesis 2 (i.e. unpaired t-tests, α = 0.01); panel (**b**) shows results of hypothesis 3 (i.e. post-hoc unpaired t-tests, α = 0.0005). * p<0.01, ** p<0.001, *** p<0.00001. P_1_ indicates the p-value of the first cluster during the stance phase, P_2_ the second cluster, etc.

**Fig 11:**
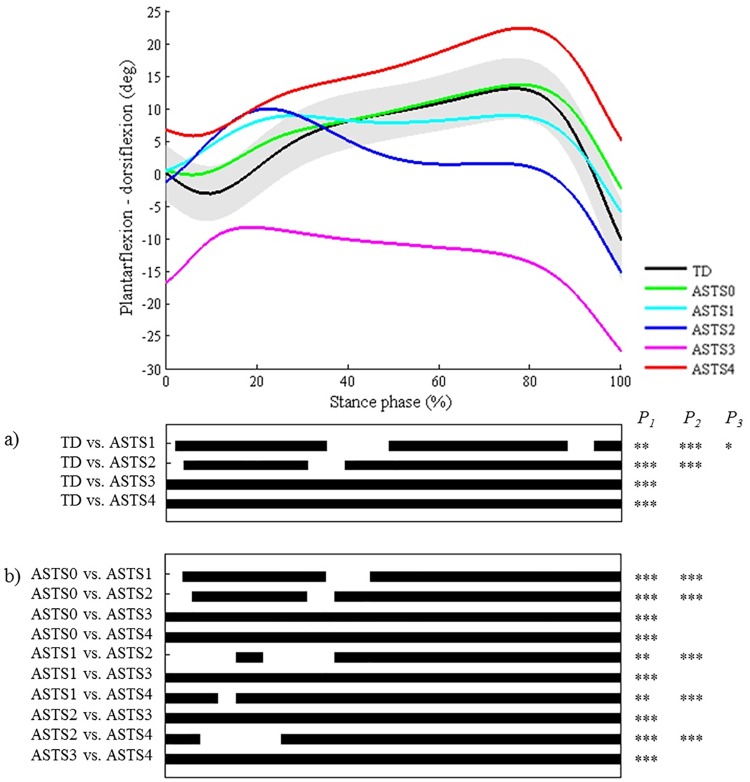
Ankle during stance in the sagittal plane (ASTS). Top graph shows the mean kinematic angle of TD gait and of each consensus-based pattern of the ankle during stance in the sagittal plane. Black bars indicate significant gait phases during which the SPM{t} statistic exceeded the critical threshold. Panel (**a**) shows results of hypothesis 2 (i.e. unpaired t-tests, α = 0.01); panel (**b**) shows results of hypothesis 3 (i.e. post-hoc unpaired t-tests, α = 0.001). * p<0.01, ** p<0.001, *** p<0.00001. P_1_ indicates the p-value of the first cluster during the stance phase, P_2_ the second cluster, etc.

**Fig 12:**
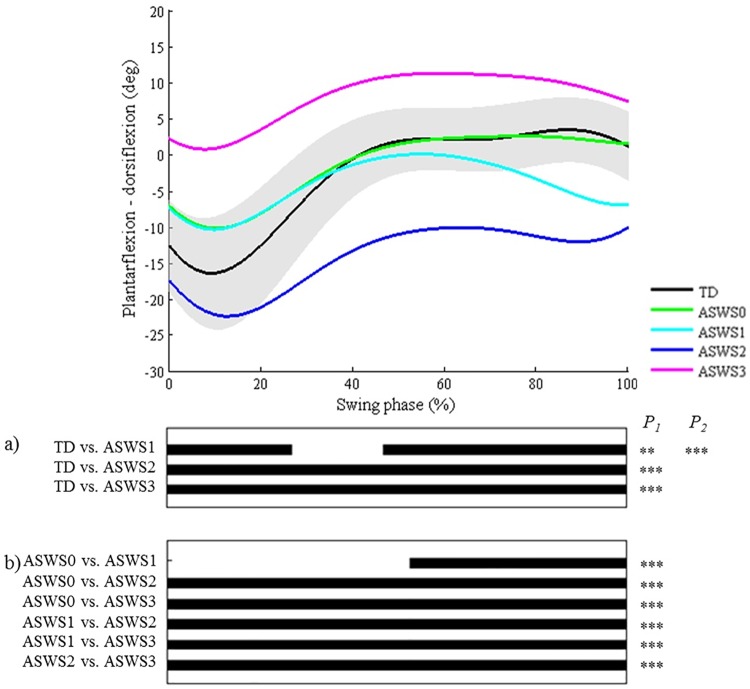
Ankle during swing in the sagittal plane (ASWS). Top graph shows the mean kinematic angle of TD gait and of each consensus-based pattern of the ankle during swing in the sagittal plane. Black bars indicate significant gait phases during which the SPM{t} statistic exceeded the critical threshold. Panel (**a**) shows results of hypothesis 2 (i.e. unpaired t-tests, α = 0.01); panel (**b**) shows results of hypothesis 3 (i.e. post-hoc unpaired t-tests, α = 0.002). * p<0.01, ** p<0.001, *** p<0.00001. P_1_ indicates the p-value of the first cluster during the swing phase, P_2_ the second cluster.

**Fig 13:**
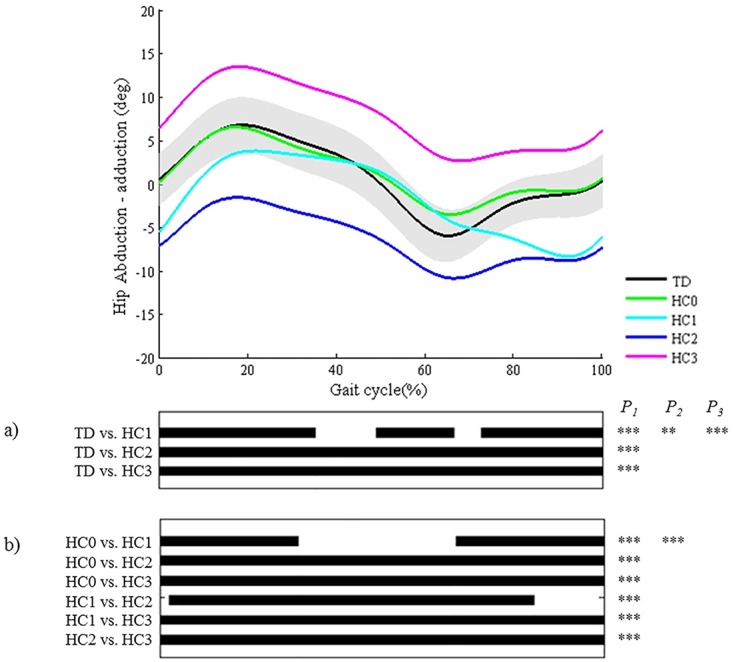
Hip in the coronal plane (HC). Top graph shows the mean kinematic angle of TD gait and of each consensus-based pattern of the hip in the coronal plane. Black bars indicate significant gait phases during which the SPM{t} statistic exceeded the critical threshold. Panel (**a**) shows results of hypothesis 2 (i.e. unpaired t-tests, α = 0.01); panel (**b**) shows results of hypothesis 3 (i.e. post-hoc unpaired t-tests, α = 0.002). * p<0.01, ** p<0.001, *** p<0.00001. P_1_ indicates the p-value of the first cluster during the gait cycle, P_2_ the second cluster, etc.

As regards the joint motion patterns of the **hip, knee (during stance/swing), and ankle (during stance/swing) in the sagittal plane and the hip in the coronal plane** (n = 29), statistical analyses identified every location during the gait cycle which was described in the classification rules (Figs 8–13). However, SPM also identified at least one joint motion pattern for each of these joints that markedly differed from TD gait (>3°) during a phase of the gait cycle that was not incorporated in the pattern definitions. Firstly, for the **hip in the sagittal plane**, ‘hip extension deficit’ (HS1), which is defined based on stance phase kinematic deviations in the sagittal plane, also presented excessive hip flexion during 80–100% of swing phase (p = 0.00004; Fig 8). Secondly, the **knee patterns during swing** are defined based on an abnormal peak flexion angle. In addition to this feature, these patterns presented with insufficient knee extension during the second half of swing phase (all p<0.00001; Fig 9). In addition, for the **knee patterns during stance**, the patterns ‘increased flexion in midstance and internal flexion moment present’ (KSTS5) and ‘increased flexion in midstance and internal extension moment present’ (KSTS6) are identical in terms of kinematic deviations (i.e. ‘increased knee flexion in midstance’). On top of excessive knee flexion in midstance, both patterns were observed with significantly increased knee flexion compared to TD gait over the entire stance phase (both p<0.00001; Fig 10). Subsequently, the pattern ‘increased knee flexion at initial contact’ (KSTS1), was further observed to have significantly increased knee flexion between 0–71% of stance phase (p<0.00001). The pattern ‘increased flexion at initial contact and earlier knee extension movement’ (KSTS2) additionally showed significantly increased knee flexion between 53–92% of stance (p = 0.00001). However, the difference between this pattern and TD gait during this phase was small (≤3°). Thirdly, the **ankle patterns during stance** representing a ‘horizontal’ or ‘reversed second ankle rocker’ (ASTS1 and ASTS2) additionally presented with significantly increased dorsiflexion during loading response compared to TD gait (both p<0.001; Fig 11). Furthermore, the patterns ‘horizontal second ankle rocker’ (ASTS1), ‘reversed second ankle rocker’ (ASTS2), and ‘calcaneus gait’ (ASTS4) differed from TD gait during pre-swing (all p<0.01). Regarding the **ankle patterns during swing**, the pattern ‘insufficient preposition in terminal swing’ (ASWS1), also showed insufficient plantarflexion between 0–27% of swing (p = 0.00083; Fig 12). Fourthly, in the **hip in the coronal plane**, the joint motion pattern ‘excessive hip abduction during swing’ (HC1), further showed excessive abduction between 0–35% of the gait cycle (p<0.00001) and slightly increased (≤3°) adduction between 49–67% of the gait cycle (p = 0.00031; Fig 13) compared to TD gait.

**Fig 14:**
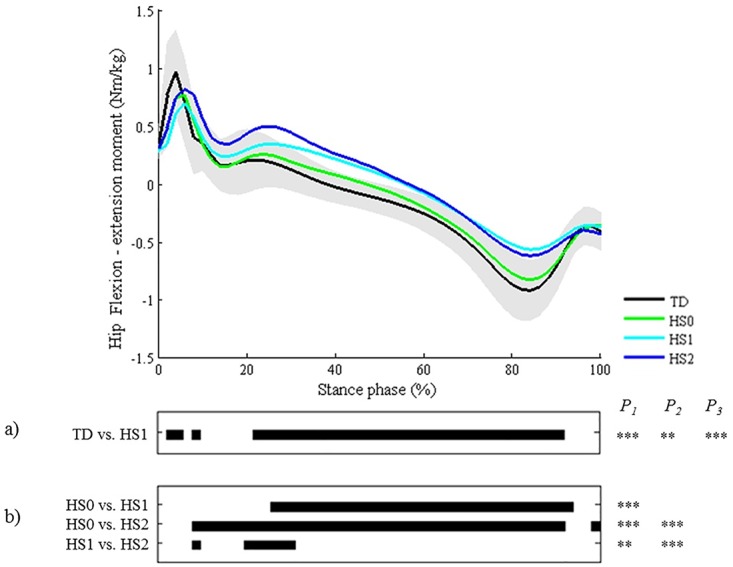
Hip in the sagittal plane (HS). Top graph shows the mean kinetic angle of TD gait and of each consensus-based pattern of the hip in the sagittal plane. Black bars indicate significant gait phases during which the SPM{t} statistic exceeded the critical threshold. Panel (**a**) shows results of hypothesis 2 (i.e. unpaired t-tests, α = 0.01); panel (**b**) shows results of hypothesis 3 (i.e. post-hoc unpaired t-tests, α = 0.003). * p<0.01, ** p<0.001, *** p<0.00001. P_1_ indicates the p-value of the first cluster during the stance phase, P_2_ the second cluster, etc.

**Fig 15:**
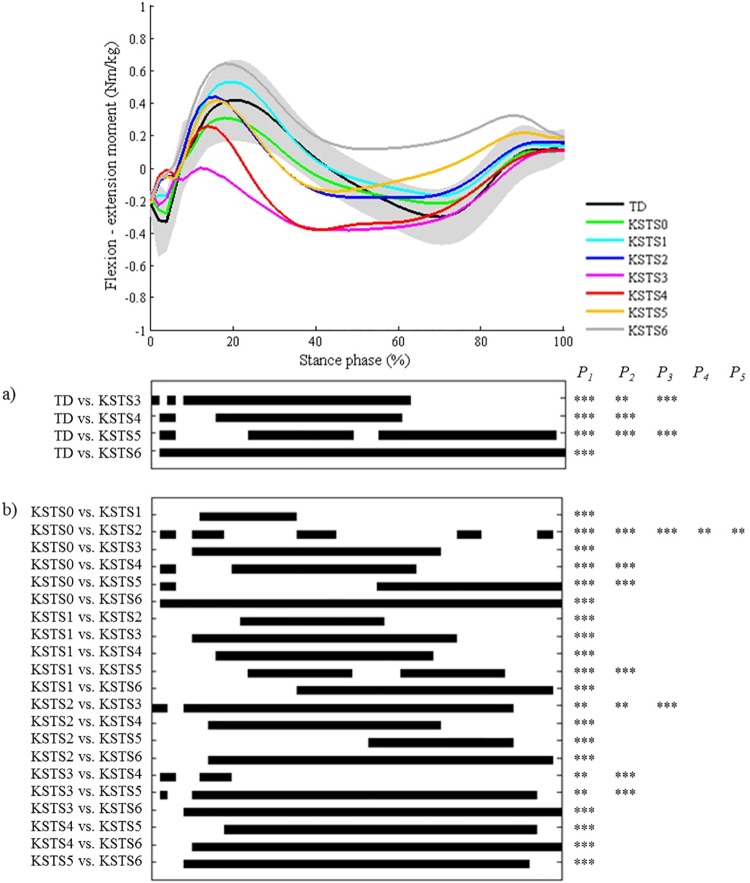
Knee during stance in the sagittal plane (KSTS). Top graph shows the mean kinetic angle of TD gait and of each consensus-based pattern of the knee during stance in the sagittal plane. Black bars indicate significant gait phases during which the SPM{t} statistic exceeded the critical threshold. Panel (**a**) shows results of hypothesis 2 (i.e. unpaired t-tests, α = 0.01); panel (**b**) shows results of hypothesis 3 (i.e. post-hoc unpaired t-tests, α = 0.0005). * p<0.01, ** p<0.001, *** p<0.00001. P_1_ indicates the p-value of the first cluster during the stance phase, P_2_ the second cluster, etc.

The mean kinetic curves of the patterns that contain a description of kinetic deviations (‘hip extension deficit’ (HS1), ‘knee hyperextension’ (KSTS3), ‘knee hyperextension and increased knee flexion at initial contact’ (KSTS4), ‘increased knee flexion in midstance and internal flexion moment present’ (KSTS5) and ‘increased knee flexion in midstance and internal extension moment present’ (KSTS6)) were all found to differ significantly from their respective TD joint moments (Figs 14 and 15). The locations of difference concurred with the classification rules. In addition to the expected significant findings, small (≤3°) significant locations were identified for each of those patterns during the first 15% of stance phase.

### Hypothesis 3: The kinematic and kinetic trials of the joint motion patterns at the level of each joint are different from each other in at least one part of the gait cycle

SPM ANOVAs of kinematic and kinetic trials identified significant differences between all joint motion patterns of each joint (p<0.01). Post-hoc SPM t-tests indicated that the patterns at the level of each joint were found to be significantly different from the other joint motion patterns, on average throughout 91% of the gait cycle (or of stance/swing phase; Figs 3–15). Only ‘decreased pelvic anterior tilt’ (PS4) and ‘decreased pelvic anterior tilt and increased range of motion’ (PS5) did not differ significantly from each other throughout the gait cycle (Fig 5).

## Discussion

This study examined the content validity of a recently published gait classification for children with spastic CP. The purpose was to provide objective evidence for the existence of joint motion patterns in CP, which were developed and subjectively defined by an expert panel via a consensus study [11]. SPM was used to analyze a large database of kinematic and kinetic trials that were classified by clinicians to investigate three hypotheses.

**The first hypothesis** assumed that the patterns with ‘no or minor gait deviations’ at the level of each joint, would not differ from the gait pattern of TD children. This hypothesis could only be confirmed for the pelvis in the transverse plane.

Since the pattern with minor gait deviations differed from TD gait for all other joints, the data suggest that common gait deviations in CP were not included in the classification, which would threaten its content validity. However, careful inspection of the results revealed two interesting findings, mostly suggesting that all relevant information is included in the pattern definitions. First, for most joints, the deviations from the mean angle of TD gait were less than 3°. It can therefore be assumed that these differences are clinically of less relevance, especially when also taking into account possible inter-therapist or inter-session measurement errors [16]. Secondly, the marked deviations (>3°) that were observed for the hip in the sagittal plane and the knee during swing phase were already incorporated in the other pathological joint motion patterns of these respective joints. For example, the observed increased hip flexion in the sagittal plane during 0–61% and 74–100% of the gait cycle refer to areas of the gait cycle that are incorporated in the patterns ‘hip extension deficit’ (HS1) and ‘continuous excessive hip flexion’ (HS2), and a patient will be classified as such if hip flexion markedly increases. Similarly, regarding insufficient knee extension during terminal swing, one could argue that this important clinical information is already sufficiently represented in the knee patterns during stance that include the feature ‘increased knee flexion at initial contact’ (KSTS1, 2, and 4).

The only results which could indicate that relevant clinical information was not included in the classification were found for the patterns of the ankle during stance and swing. Statistically significant and large differences were identified during the first and third ankle rocker, as well as during early swing. Deviations in these locations of the gait cycle additionally appeared in the results for the second and third hypotheses as being discriminatory between different joint motion patterns. Specific kinematic deviations related to the first and third ankle rockers are currently not included in the pattern definitions of the studied classification, nor were they included in previously reported classifications [6–10]. It should be further investigated to what extent these locations can help improve the current patterns definitions.

**The second hypothesis** assumed that all other pathological joint motion patterns differed significantly from the gait pattern of TD children in the key locations of the gait cycle that were indicated in the pattern definitions by the experts. A first general conclusion from the results is that for each pattern, all key locations that were originally included in the classification rules were indeed highlighted as significant areas by the SPM analysis. Secondly, we could conclude that on several occasions, additional significant locations were indicated by SPM analysis during which patterns also differed from TD gait, even though these locations were not described in the Delphi study. These results could be used to further refine some pattern definitions, for example for some of the patterns of the knee during stance (a) and swing (b) in the sagittal plane. (a) Regarding the knee pattern during stance, it was clear that patients, who fulfill the current criteria of excessive flexion during midstance, will also show excessive knee flexion during the remainder of stance (Fig 10). The kinematic deviations of the patterns ‘increased knee flexion in midstance and internal flexion moment present’ (KSTS5) and ‘increased knee flexion in midstance and internal extension moment present’ (KSTS6) might therefore be redefined as ‘continuously excessive knee flexion during stance’, similar to the crouch pattern that was defined by Sutherland et al. [19]. The results related to the third hypothesis (Fig 10) indicated that ‘increased knee flexion in midstance and internal extension moment present’ (KSTS6) also showed significantly higher knee flexion than ‘increased knee flexion in midstance and internal flexion moment present’ (KSTS5) between 10–67% of the stance phase, even though the definitions of these patterns in terms of kinematic deviations were identical in the consensus study [11]. The mean angle of KSTS6 reaches over 30° of knee flexion whereas the mean angle of KSTS5 does not. This information could help clinicians distinguish between these two patterns for patients that do not have kinetic data or trunk kinematics available, as trunk position will likely be an important factor influencing the generated knee moment during stance. (b) All knee patterns during swing were characterized by insufficient knee extension during terminal swing compared to TD gait. In addition, the results related to the third hypothesis (Fig 9) clearly highlighted that all patterns without the feature ‘delayed peak knee flexion’ (KSWS0-2-4) reached a similar knee flexion angle during terminal swing, which was significantly lower than the angles of all patterns with the feature ‘delayed peak knee flexion’ (KSWS1-3-5), but also approximately 10° higher than the angle of TD gait. If there is doubt about whether or not the peak knee flexion during swing is delayed, the knee angle during terminal stance could support the final choice. Previously, Rha et al. have also shown significant correlations between the timing of peak knee flexion during swing and knee flexion angle at initial contact.[20]

The **third hypothesis** assumed that all pathological patterns at the level of each joint are different from each other in at least one part of the gait cycle. This hypothesis was confirmed for all patterns, apart from two pelvic patterns in the sagittal plane: ‘decreased anterior tilt’ (PS4) and ‘decreased anterior tilt and increased range of motion’ (PS5). The low observed frequency of these patterns (1.3% and 1.1% respectively) in this study might have limited the power of the SPM analysis to detect significant differences between both patterns. Also in literature, decreased pelvic tilt was not often described in CP gait classifications. The usefulness of these two patterns in the classification should therefore be questioned. Only Rodda et al. [21] have mentioned decreased tilt as a possible feature of the Type IV gait pattern, which represents patients with severe crouch gait (i.e. excessive hip and knee flexion as well as excessive ankle dorsiflexion).

Regarding the statistical analyses, SPM unpaired t-tests were used for the first two hypotheses and SPM one-way-ANOVA was used to test the third. Alternatively, an SPM one-way-ANOVA could have been performed for each joint, including both the CP joint motion patterns and the TD gait trials. The post-hoc SPM t-tests would essentially constitute all comparisons that are reported in the present study, except that the critical threshold would be calculated based on a lower α-level because of the Bonferroni correction. To test whether this choice of statistics would have affected the conclusions, these analyses were also performed. Results showed that probabilities were lower and for several between-group comparisons the width of the clusters was slightly more narrow (generally for 2–4% of the gait cycle), but never to the extent that it would change the interpretation of the results. A limitation of this study is that the assumption of equal variance between all pathological patterns and TD gait could have been violated. It is possible that slightly higher critical thresholds would have been identified if corrections for unequal variances would have been performed, but this feature is challenging to be defined and was not available using the current SPM code for Matlab. Slightly stricter critical thresholds are not likely to alter the general conclusions of this study (cfr. supra), as the probability of most critical thresholds was very low (p<0.00001). A possible effect could be that some differences between TD gait and the patterns ‘no or minor gait deviations’ of the foot progression angle, ankle during swing phase in the sagittal plane, and knee during stance phase in the sagittal plane might have been undetected, as the mean angles between these patterns and TD gait were smaller than 3° and probabilities for the supra-threshold clusters of these analyses were relatively close to 0.01 (Fig 2). Although all trials were considered independently, a potential learning effect could not be excluded as raters could not be blinded to patient identification. However, previous repeatability analyses suggested that this most likely did not influence the results [22].

## Conclusion

The currently presented results support the content validity of the examined joint motion patterns in CP. It was found that most patterns with ‘no or minor gait deviations’ differed somewhat unexpectedly from TD gait, but differences were generally small (<3°). Further evidence demonstrated that the other pathological joint motion patterns differed from TD gait and from each other. The locations of significant difference between the patterns and TD gait coincided well with the subjective, consensus-based classification rules. Nonetheless, some additional areas, which were not included within the pattern definitions of the consensus study, were also highlighted by the SPM analysis. Based on these results, suggestions to improve current pattern definitions were made. The results further suggest that algorithms, which could automate this classification [13], are likely to be successful. Future research should establish to what extent the patterns are responsive to treatment and how they could be incorporated in the clinical reasoning process.

## Supporting Information

S1 Checklist (PDF)

S1 Table Final overview of joint patterns and their criteria after last Delphi survey. (PDF)
