# Supplementary material for: Statistical Parametric Mapping to Identify Differences between Consensus-Based Joint Patterns during Gait in Children with Cerebral Palsy
Source: PLoS One. 2017 Jan 12;12(1):e0169834. doi: 10.1371/journal.pone.0169834 (PMC5231378; doi:10.1371/journal.pone.0169834)
Supplement: S1 Table — (PDF) [file pone.0169834.s002.pdf]

Table S1. Final overview of joint patterns and their criteria after last Delphi survey

		Joint pattern	Full description and criteria
**Sagittal plane**	**Pelvis**	Normal pelvic posture/motion	
		Increased range of motion	
		Increased pelvic anterior tilt on average	
		Increased pelvic anterior tilt + increased range of motion	
		Decreased pelvic tilt (posterior tilt) on average	
		Decreased pelvic tilt (posterior tilt) + increased range of motion	
	**Hip**	Normal hip motion	
		Hip extension deficit	At least two of the following characteristics: (1) decreased hip extension in stance, (2) decreased hip range of motion in stance, (3), delayed timing of zero hip moment or decreased hip flexion moment
		Continuous excessive hip flexion	Excessive hip flexion throughout at least 90% of the gait cycle AND hip flexion angle continuously above 0°
	**Knee**	Normal knee in stance	
	**(during stance)**	Increased knee flexion at initial contact	
		Increased knee flexion at initial contact + earlier knee extension movement	
		Knee hyperextension	At least two of the following characteristics: (1) increased knee extension (to knee hyperextension) in mid- or late stance, (2) earlier knee extension movement in stance, (3) excessive knee flexion moment in mid- or late stance
		Knee hyperextension + increased knee flexion at initial contact	Increased knee flexion at initial contact AND at least 2 out of 3 features: (1) increased knee extension (to knee hyperextension) in mid- or late stance, (2) earlier knee extension movement in stance, (3) excessive knee flexion moment in mid- or late stance
		Increased knee flexion in midstance + internal knee flexion moment present	Increased knee flexion in midstance: no normal knee angle in extension in midstance AND internal knee flexion moment is present for at least 1/3rd of stance phase
		Increased knee flexion in midstance + internal knee extension moment present	Increased knee flexion in midstance: no normal knee angle in extension in midstance AND internal knee extension moment is present for at least 2/3rd of stance phase

(Table S1. Continued)

	**Knee**	Normal knee in swing	
	**(during swing)**	Delayed peak knee flexion	
		Increased peak knee flexion	
		Increased + delayed peak knee flexion	
		Decreased peak knee flexion	
		Decreased + delayed peak knee flexion	
	**Ankle**	Normal ankle in stance	
	**(during stance)**	Horizontal second ankle rocker	Horizontal pattern of second ankle rocker from loading response (10%) to start push-off (slope <5°)
		Reversed second ankle rocker	Descending pattern of second ankle rocker from loading response (10%) to start push-off (slope ≥ -5°)
		Equinus	Continuous plantarflexion (x<0°) throughout stance
		Calcaneus gait	Increased slope towards dorsiflexion during stance OR a peak ≥ 20° of dorsiflexion
	**Ankle**	Normal ankle in swing	
	**(during swing)**	Insufficient prepositioning in terminal swing	Ankle plantarflexion at initial contact at the end of the gait cycle, which is greater than normal values
		Continuous plantarflexion in swing (drop foot)	Excessive plantarflexion for most of the swing phase, at least hindering foot clearance around 90% of the gait cycle AND ankle plantarflexion at initial contact at the end of the gait cycle, which is greater than normal values
		Excessive dorsiflexion in swing	Increased dorsiflexion in swing for at least 1/3rd of the swing phase
**Coronal plane**	**Pelvis**	Normal pelvic posture/motion	
		Increased pelvic range of motion	
		Continuous pelvic elevation (up)	
		Continuous pelvic depression (down)	
	**Hip**	Normal hip motion	
		Excessive hip abduction in swing	
		Continuous excessive hip abduction	
		Continuous excessive hip adduction	

(Table S1. Continued)

**Transverse plane**	**Pelvis**	Normal pelvic posture/motion	
		Increased pelvic range of motion	
		Excessive pelvic external rotation during the gait cycle	
		Excessive pelvic internal rotation during the gait cycle	
	**Hip**	Normal hip motion	
		Excessive hip external rotation during the gait cycle	
		Excessive hip internal rotation during the gait cycle	
	**Foot**	Normal foot progression angle	
		Outtoeing	Excessive external foot progression on average during stance
		Intoeing	Excessive internal foot progression on average during stance

John Wiley and Sons, © 2015 Mac Keith Press; Reprinted by permission[1].

[1] Nieuwenhuys A, Õunpuu S, Van Campenhout A, Theologis T, De Cat J, Stout J, et al. Identification of joint patterns during gait in children with cerebral palsy: A Delphi consensus study. Dev Med Child Neurol 2016;58:306–13. doi:10.1111/dmcn.12892.
